# Microleakage evaluation of silorane based composite versus methacrylate based composite

**DOI:** 10.4103/0972-0707.71649

**Published:** 2010

**Authors:** Roula Al-Boni, Ola M Raja

**Affiliations:** Department of Operative Dentistry, School of Dentistry, Damascus University, Damascus, Syria

**Keywords:** Microleakage, polymerization shrinkage, silorane, stress

## Abstract

**Aim:**

Our study aimed to analyze the microleakage of silorane-based composite in comparison to two homologous methacrylate-based composites.

**Materials and Methods:**

Standardized class I cavities (4 × 2.5 × 3 mm) were prepared on extracted human premolars and randomly assigned into three groups (*N* = 15) as follows: Group A, Filtek P90 (silorane) with its dedicated adhesive system (P90 system adhesive); Group B, Adper SE Plus with Filtek Z250; Group C, Peak SE with Amelogen Plus. The teeth were subjected to thermocycling regime (200×, 5–55°C) and dye penetration of tooth sections were evaluated following 30 minute immersion in 2% Methylene Blue dye. Statistical analysis was performed with Kruskal-Wallis and Mann-Whitney *U* test at 95% significance level.

**Results:**

Silorane exhibited significantly decreased microleakage compared with any other resin based composite (RBC). The cavities restored with Amelogen Plus displayed nonsignificantly higher microleakage than with Filtek Z250.

**Conclusion:**

Although all of the restorative systems had microleakage, silorane technology showed less microleakage comparable to clinically successful methacrylate-based composite. This will improve the clinical performance and extend the composite durability.

## INTRODUCTION

Resin composite materials have improved greatly since their introduction and there is general shifting away from amalgams toward composite resins.[1] Although composites are now the material of choice for most restorations,[2] their polymerization shrinkage remains a problem.[3,4] The contraction stress associated with this shrinkage can cause debonding at the composite/tooth interface and can contribute to postoperative sensitivity, enamel fracture, recurrent caries, marginal staining and eventual failure of the restoration.[4]

Slowing down the composite polymerization rate,[5] placing thicker adhesive layers under the composite,[6] and using an incremental placement technique[7] or low-modulus intermediate layers[8] are just some manipulations suggested to reduced shrinkage stress for a given composite material at the composite/tooth interface. Other factors also can be considered, for example, the cavity configuration factor (C-factor) which describes the ratio of bonded surfaces to unbonded surfaces in a restoration.[9] With the use of bonded shrinking polymeric materials,[10] high C-factors are accompanied by greater internal stresses.

For dental purposes, siloranes, a new class of ring-opening monomers, were synthesized to overcome the problems related to polymerization shrinkage.[1,11] This new type of monomer is obtained from the reaction of oxirane and siloxane molecules. The volumetric shrinkage of a silorane-based composite was determined to be 0.99 vol. % using the Archimedes method.[12] The novel resin is also considered to have combined the two key advantages of the individual components: low polymerization shrinkage due to oxirane monomers and increased hydrophobicity due to the presence of the siloxane species in its composition. The mechanism of compensating stress in this new system is achieved by the opening and extending of the oxirane rings during polymerization to compensate volume reduction by monomers packing.[12]

Apart from the predominant radical polymerization initiation in conventional methacrylate resin based composites (RBCs), the silorane composite polymerizes by a cationic process, which is insensitive to oxygen. The silorane-based composite revealed decreased water sorption, solubility and associated diffusion coefficient compared with conventional methacrylate RBCs.[13] Moreover, Palin *et al*.[14] showed that cusp deflection caused by polymerization shrinkage and the microleakage was significantly lower for a silorane material than for methacrylate RBCs (Filtek Z250, 3M ESPE, St. Paul, MN, USA). Previous studies (conducted by the manufacturer) have shown a significantly improved marginal integrity on both enamel and dentin of silorane RBCs compared with methacrylate RBCs.[15] Weakening of the adhesive resin due to thermo-mechanical loading is an important issue in restorative dentistry. Studies suggest that thermocycling could accelerate deterioration of the dentin/restoration interface.[16] Therefore, this research compared the microleakage of a low-shrinkage resin composite Filtek P90 (Silorane, 3M ESPE) and two hybrid resin composites Filtek Z250 (3M ESPE) and Amelogen Plus (Ultradent) by means of dye penetration after thermocycling.

## MATERIALS AND METHODS

Forty-five extracted intact upper premolars were selected. The teeth were scaled with ultrasonic, cleaned with pumice by a rotary brush and stored in 0.5% chloramine T at 4°C for 1 week and then in distilled water until use.

The teeth received standardized class I cavity preparations, approximately 4 mm in length, 2.5 mm in width and 3 mm in depth, using Cavity Coping Machine that prepares cavities of the same size, similar to that prepared in a metal model. We used diamond burs (#837 Komet Gebr., Brasseler, Lemgo, Germany) in a high speed handpiece, under constant water irrigation for all cavities (the bur is changed every five preparations). The cavosurface margins were prepared at 90°.

The teeth were randomly divided into three groups (*N* = 15) according to the restorative material used, as follows.

Group A: Low shrinkage resin composite Filtek P90 (lot 9BY, 3M ESPE, St.Paul, MN, USA) with LS System Adhesive Primer and Bond (lot 8BA, 3M ESPE, St.Paul, MN, USA) were used. The tooth was blot-dried, leaving a moist structure. The P90 Primer was applied using a microbrush with agitation for 15 seconds, gently air-dried, then light-cured for 10 seconds, then the P90 Bond was applied followed by a gentle stream of air, and light-cured for 10 seconds. The composite was applied in two wedge shaped incremental layers. The first composite increment was placed on the pulpal floor and buccal wall, then light activated according to the manufacturer’s instructions for 20 seconds. Second composite increment was placed obliquely on the palatal wall and extended on the occlusal surface, and then light-cured. Immediately after the filling procedure, the restorations were finished and polished with flexible disks Compo System (Komet Gebr.). Finishing and polishing were done under simultaneous water cooling to avoid drying out of the teeth.

Group B : Filtek Z250 resin composite (lot 8CP, 3M ESPE, St.Paul, MN, USA) with Adper SE Plus Self-Etch Adhesive (lot 8BJ, 3M ESPE, St.Paul, MN, USA) were used. Pink color Liquid A was applied using microbrush, and then the brush was disposed (acting as a guide for achieving full coverage when applying the adhesive). Once yellow Liquid B is applied with rubbing motion for 20 seconds, the pink color disappears. After passing gentle air stream, Liquid B was re-applied and then light-cured for 10 seconds. The composite Filtek Z250 was applied using the same protocol as described before.

Group C : Amelogen Plus (lot B3VZ3, Ultradent, South Jordan, UT, USA) with Peak Self-Etch Adhesive System (lot B3V25, Ultradent) were used. Peak SE primer was vigorously scrubbed onto the tooth surface for 15 seconds and the excess primer was dispersed with a stream of air. Peak SE bonding agent was applied and dispersed with a weak stream of air for 10 seconds, followed by polymerization for 10 seconds. The Amelogen Plus composite was applied using the same protocol as described above.

All composite increments were light-cured using an Elipar Freelight 2 light-curing unit (3M ESPE, Seefeld, Germany) at a power density of 1000 mW/cm^2^ for 20 seconds in a continuous mode, while all adhesive systems were light-cured using the same light-curing unit, power density and mode for 10 seconds. The light intensity was constantly monitored by its integrated radiometer. All adhesive systems used in this study were two steps self-etch adhesive systems and all the composites were micro-hybrid A2 shade.

After specimens were stored in distilled water for 24 hours at 37°C, the teeth were subjected to a thermocycling regime (200 cycles) with a dwelling time of 30 seconds and transfer time of 5 seconds, between 5°C and 55°C. For microleakage evaluation, the root apices were sealed with sticky wax, and the root and crown surfaces of the teeth were sealed with two coats of nail varnish, except for 1 mm around the restoration margins. The teeth were then immersed in 2% Methylene Blue dye (pH = 7) for 30 minutes,[17] washed and dried. Next, the teeth were sectioned mesio-distally into two slabs using a slow-speed diamond saw (Komet Gebr.). Four sites per tooth (cavosurface angle to pulpal floor from mesial and distal walls for each slab) were examined under an optical stereomicroscope at 20× magnification and dye penetration was scored as described in Table 1.

**Table 1:** In-depth dye penetration scores

Score	Definition
0	No dye penetration at all
1	Dye penetration up to one-third of the vertical cavity wall
2	Dye penetration up to two-thirds of the vertical cavity wall
3	Dye penetration up the pulpal floor
4	Dye penetration extends in the pulpal floor

The median of the scores was subjected to statistical analysis using the non-parametric Kruskal-Wallis analysis of variance test and the Mann-Whitney test at a 95% significance level.

## RESULTS

Median leakage scores and mean ranks for each group are listed in Table 2. Kruskal-Wallis test indicated significant difference in one group at least (*P* < 0.05). Mann-Whitney *U* test was used to make a pairwise comparison between the three studied groups; it shows significant difference between silorane and the two other methacrylate RBCs [Table 3].

Referring to mean rank values, we conclude that microleakage scores in Filtek P90 (silorane) were significantly lower than those of both RBC groups (*P* < 0.05). There is no significant difference in microleakage scores between Filtek Z250 and Amelogen Plus groups (*P* > 0.05).

**Table 2:** Mean ranks as in Kruskal-Wallis test

Variable studied	Composite type	*N*	Mean rank	Chi square	Degree of freedom	*P* value	Significant difference?
Leakage degree	Filtek P90 (silorane)	60	76.82	11.31	2	0.003	Yes
	Filtek Z250	60	95.20				
	Amelogen Plus	60	99.48				
	Total	180					

**Table 3:** Mann-Whitney *U* test exhibits significant difference between the groups

Group A	Group B	*U* value	*P* value (two-tailed)	Significant difference?
Filtek P90 (silorane)	Filtek Z250	1425.0	0.015	Yes
	Amelogen plus	1354.0	0.003	Yes
Filtek Z250	Amelogen Plus	1707.0	0.452	No

## DISCUSSION

According to Ferracane,[18] the polymerization shrinkage of dental composites is of the order of 1.5–5% which is enough to result in the development of internal stresses that might threat the durability of a composite restoration. A new resin silorane system was developed to overcome this issue.[11,12]

The present study compared the microleakage of a novel low-shrinkage resin composite to other clinically successful methacrylate resin composites. Class I cavities were used due to the high C-factor that causes greater polymerization stresses[9] as a result of restrained contraction by the large number of bonded surfaces. Microleakage evaluation is the most common method of assessing the sealing efficiency of a restorative material. Since there is no gold standard for this method, we used 2% Methylene Blue for 30 minutes as was previously used by Ernst[17] who concluded that this immersion period in this concentration had a good correlation with the marginal gaps evaluated using Scanning Electron Microscope.

All three resin composite materials used in this study had some degree of leakage. We had noticed that the percentage of score 4 in the microleakage was dominant on the specimens, which can be attributed to the high light intensity in a continuous mode, the high C-factor (5/1) of class I cavity, self-etch hydrophilic adhesive systems or the relatively high concentration of Methylene Blue dye. The thermal stresses encountered following thermocycling, resulting in failure of the adhesive system at the interface of the tooth and restoration, may have contributed to the increase in microleakage.

The results of this study show that the new low-shrink composite restorative material had significantly lesser microleakage after thermocycling than that of Amelogen Plus/Peak SE and Filtek Z250/Adper SE Plus. The significant decrease in microleakage of the cavities restored with the Filtek P90 (silorane) compared with the other RBCs was attributed to the inherent ring opening polymerization of the silorane monomers which can compensate the volume reduction as the molecules come closer to each other compared to the radical polymerization of the other RBCs, which is liner polymerization, manifested as a reduction in polymerization shrinkage stress at the tooth/restoration interface. It is also hypothesized that since silorane technology provides lower polymerization shrinkage and related polymerization stress than methacrylate RBCs,[12] it should be able to withstand thermocycling fatigue at the interface better than the microhybrid composites tested in this study. So, we agree with Palin, Yamazaki and Bagis[14,19,20] who had proved that the microleakage of silorane is lower than that of methacrylate based composite, after loading. We disagree with Ernst[17] who proved that the microleakage of teeth restored with silorane was similar to others restored with methacrylate composite (Tetric Ceram) With adhesive (Clearfil SE Bond) and the reason for this is that the author had used an all-in-one (7th generation) experimental bond of silorane previously produced by the company and we used the new bond produced with silorane which is two-steps, two-components bond (6th generation), thereby giving different results.

The nonsignificant differences in microleakage of cavities restored with Amelogen Plus and Filtek Z250 may be associated with the similarities in the methacrylate chemistry and the utilization of self-etch adhesive systems for both methacrylate RBCs. As resin composites still undergo contraction stress over time and damage of marginal sealing after water storage,[21] long-term data are still necessary. In addition, it has been demonstrated that the association of mechanical loading with thermal cycling may significantly increase leakage values.[22] Thus, further studies evaluating the influence of storage and mechanical loading on microleakage are required.

## CONCLUSIONS

Within the limitations of this study, the authors conclude that: none of the restorative systems tested totally prevented microleakage; the low-shrink silorane system had significantly lesser microleakage than Amelogen Plus/Peak SE and Filtek Z250/Adper SE Plus and there is no significant difference between both methacrylate RBCs.
